# TGF-β2, EGF, and FGF21 Growth Factors Present in Breast Milk Promote Mesenteric Lymph Node Lymphocytes Maturation in Suckling Rats

**DOI:** 10.3390/nu10091171

**Published:** 2018-08-27

**Authors:** Paulina Torres-Castro, Mar Abril-Gil, María J. Rodríguez-Lagunas, Margarida Castell, Francisco J. Pérez-Cano, Àngels Franch

**Affiliations:** 1 Physiology Section, Department of Biochemistry and Physiology, Faculty of Pharmacy and Food Science, University of Barcelona, 08028 Barcelona, Spain; mtorreca29@alumnes.ub.edu (P.T.-C.); mariadelmar.abril@ub.edu (M.A.-G.); mjrodriguez@ub.edu (M.J.R.-L.); margaridacastell@ub.edu (M.C.); angelsfranch@ub.edu (À.F.); 2 Nutrition and Food Safety Research Institute (INSA·UB), 08921 Santa Coloma de Gramenet, Spain

**Keywords:** growth factors, breast milk, immunonutrition, cytokines, lymphocytes

## Abstract

Breast milk, due to its large number of nutrients and bioactive factors, contributes to optimal development and immune maturation in early life. In this study, we aimed to assess the influence of some growth factors present in breast milk, such as transforming growth factor-β2 (TGF-β2), epidermal growth factor (EGF), and fibroblast growth factor 21 (FGF21), on the immune response development. Newborn Wistar rats were supplemented daily with TGF-β2, EGF, or FGF21, throughout the suckling period. At day 14 and 21 of life, lymphocytes from mesenteric lymph nodes (MLNs) were isolated, immunophenotyped, and cultured to evaluate their ability to proliferate and release cytokines. The main results demonstrated that supplementation with TGF-β2, EGF, or FGF21 modified the lymphocyte composition in MLNs. At day 14, all supplementations were able to induce a lower percentage of natural killer (NK) cells with the immature phenotype (CD8^+^), and they reduced the CD8αα/CD8αβ ratio at day 21. Moreover, the cytokine pattern was modified by the three treatments, with a down regulation of interleukin (IL)-13 secretion. These results showed the contribution of these growth factors in the lymphocytes MLNs immune maturation during the neonatal period.

## 1. Introduction

At time of birth, the intestine is immature, not only anatomically, but also metabolically and immunologically [1,2,3]. Intestinal development is a key process in early life because it includes important functions related to growth and survival. It is important to develop mechanisms to digest and absorb nutrients in an efficient way [4]. Moreover, the intestine begins hosting the gut microbiota and establishing appropriate host immune responses against pathogens [2]. The intestinal maturation process can be conditioned through synergy of several factors, such as genetics, microbial colonization, and nutrition [1,2,3].

Breast milk is the gold standard to feed the newborn because it includes a rich number of components, which are essential for optimal growth and development [5]. It also contains a high number of bioactive factors, which participate in the immune maturation process of infants [2,6]. Among these components, immunoglobulins (Igs), cytokines, and growth factors (GFs) have an important role [7,8,9]. In this sense, the effects of several GFs present in maternal milk that promote intestinal maturation have been described, although their impact on immune development is still unclear [9].

On the one hand, the transforming growth factor-β (TGF-β) family members have multifunctional actions involved in maintaining intestinal homeostasis, regulating inflammation, and allergy development [10,11,12]. TGF-βs act on different types of leukocytes, to control the initiation and resolution of immune responses through the recruitment, activation, and survival of cells [12,13]. TGF-β2 is the predominant isoform present in human and rat breast milk; it reaches the neonatal intestine where, at birth, endogenous production is low and increases towards weaning [3,10,12]. In infants, breast milk TGF-βs play an important role in developing immune response and promoting oral tolerance development [12].

On the other hand, one of the most abundant GFs in breast milk is the epidermal growth factor (EGF), the concentration of which is 500 times more than other GFs present in breast milk [14]. Its concentration is very high in colostrum and it decreases significantly during suckling, both in human and rodent milk, suggesting that EGF plays a role in the promotion of early neonatal intestinal growth [2,15]. In fact, EGF is a key intestinal regulator in protecting intestinal barrier integrity, essential for the absorption of nutrients and the exclusion of pathogens, in both humans and animals [14,16,17]. EGF is a polyfacetic molecule, which acts by regulating different processes, such as cell growth, survival, migration, apoptosis, proliferation, and differentiation. In early life, milk EGF seems to be one of the crucial components involved in the prevention of necrotizing enterocolitis (NEC) [1,14].

EGF and TGF-β together with the immunosuppressive interleukin (IL)-10, also present in breast milk, are involved in the functional development of the gastrointestinal mucosa, tolerance acquisition, and inflammation downregulation in damaged intestinal cells [6,7].

In recent years, new components present in breast milk have been discovered. This is the case of the fibroblast growth factor 21 (FGF21), which belongs to the hormone-like subgroup within the FGF superfamily, and has been found in rodent and human milk [16]. The FGF21 present in milk, seems to be involved in local actions in the neonatal intestine. It is a highly active pleiotropic factor, involved in multiple aspects of metabolism through a variety of mechanisms, where it regulates both the expression and activity of digestive enzymes, and the synthesis and release of several intestinal hormone factors [16,18]. All these effects make FGF21 a good candidate to be studied, due to its possible contribution to neonatal intestinal function.

Overall, because TGF-β2, EGF, and FGF21 are biologically active factors found in breast milk and are suggested to be involved in neonatal intestine maturation; our hypothesis is that they could also have an important role in the immune development process in early life. Therefore, the main objective of the current study was to ascertain whether the effect of a daily supplementation with these compounds could promote immune response development, during the suckling period in rats. The effects of the supplementations on mesenteric lymph nodes (MLNs), an inductor site of the gut associated lymphoid tissue (GALT), were studied specifically during and at the end of the suckling period, in terms of lymphocyte phenotypic composition and on the immune functionality, such as their lymphoproliferative and cytokine production abilities.

## 2. Materials and Methods

### 2.1. Animals

Pregnant Wistar rats (G15) were obtained from Janvier Labs (Le Genest-Saint-Isle, France), and were individually housed in cages under controlled conditions of temperature and humidity in a 12:12 h light:dark cycle with access to food and water *ad libitum*. The pregnant rats were monitored daily and allowed to deliver naturally. The day after birth was reported as day 1. The studies were performed in accordance with institutional guidelines, for the care and use of laboratory animals, and were approved by the Ethical Committee for Animal Experimentation (CEEA) at the University of Barcelona (UB) and Catalonia Government (CEEA-UB Ref. 220/15, UB/DAAM 8521).

### 2.2. Dietary Supplementation

The suckling pups were unified in litters of nine pups per mother and were randomized into four groups, formed by three litters each (*n* = 27 pups/group). This sample size was required for each group, as previous studies have demonstrated the remarkable role of variability among litters [19]. The Appraising Project Office’s program, from the Universidad Miguel Hernández de Elche (Alicante) was used for such estimation, to detect statistically significant differences among groups, assuming there was no dropout rate and a type I error of 0.05 (two-sided).

Besides the reference group (REF group), three supplemented groups based on nutritional intervention were created: the transforming growth factor-β2 (TGF-β2), epidermal growth factor (EGF), and the fibroblast growth factor 21 (FGF21). All animals were identified daily, weighed, and supplemented by oral gavage with a volume of 10 mL/kg/day during the suckling period, from day 1 to day 21 of age. The suckling pups were separated from their mothers 30 min before oral administration, to allow gastric emptying. All daily handling was done at the same period of the day, to avoid modifications in biological rhythms. All actions were performed as described in previous studies in the group, References [19,20].

TGF-β2, EGF, and FGF21 groups were supplemented with recombinant human TGF-β2, recombinant rat EGF, and recombinant human FGF21 (all from Peprotech^®^, Rocky Hill, NJ, USA). The products were reconstituted according to the manufacturer’s recommendations.

The dose of TGF-β2 was 35 µg/kg/day, which was based on the amount of TGF-β2 found in the last lactation rat milk (62 ng/mL), and the milk intake by pups within 4−14 days of age [3]. The dose of EGF was 100 µg/kg/day, which had been demonstrated to be effective as a treatment in a rat model of NEC [21]. Finally, the dose of FGF21 was 5 µg/kg/day, an amount that was established in relation to TGF-β2, which has been found in a 1:10 ratio FGF21:TGF-β2 [10,16]. The REF group received a matched volume of the vehicle used for the GFs administration (1% bovine serum albumin (BSA) in phosphate buffer saline (PBS)).

### 2.3. Measurement of Growth and Development

Body weight was registered daily throughout the study. Two end points were established, at day 14 and at the end of the suckling period at day 21. At these times, prior to sacrifice, the pups were anesthetized with intramuscular ketamine (90 mg/kg; Imalgene^®^, Merial, Barcelona, Spain) and xylazine hydrochloride (10 mg/kg, Rompun^®^, Bayer, Barcelona, Spain); and body length (nose-anal) was measured. These data allowed the calculation of morphologic variables, such as the body mass index (BMI, g/cm^2^) and Lee index, for assessing obesity in rats ((g^1/3^/cm) × 1000).

### 2.4. Sample Collection and Processing

Once anesthetized, MLNs and small intestine (SI) were obtained through a ventral laparotomy. The SI was weighed, measured, and divided into three equal length portions. Gut washes (GWs) were obtained from the distal SI. Briefly, the intestine was flushed with cold PBS and cut into 5 mm pieces. The tissue was incubated with PBS (10 min, 37 °C, shaking), centrifuged (538 g, 10 min, 4 °C), and later, supernatant was collected and stored at −20 °C until Igs quantification.

### 2.5. Quantification of Intestinal IgA and IgM by ELISA

The IgA and IgM content were quantified in GWs from day 21 of study, by ELISA Quantitation Set (Bethyl Laboratories, Inc., Montgomery, MD, USA), as previously described in Reference [19]. GWs were diluted at 1:20 (IgA) and 1:10 (IgM). Data were expressed as µg/g of tissue.

### 2.6. Lymphocyte Isolation from Mesenteric Lymph Nodes

The MLNs were placed in complete culture medium, containing Roswell Park Memorial Institute (RPMI 1640, Sigma-Aldrich, St. Louis, MO, USA) 10% fetal bovine serum (FBS, Sigma-Aldrich, St. Louis, MO, USA), 1% l-glutamine (Sigma-Aldrich, St. Louis, MO, USA), 1% penicillin streptomicin (PenStrep; Sigma-Aldrich, St. Louis, MO, USA), and 0.05 mM 2-β-mercaptoethanol (Merck, Darmstadt, Germany). MLNs lymphocytes were obtained in sterile conditions by passing the tissue through a cell strainer (40 µm, BD Biosciences, San Diego, CA, USA). The cell suspensions were centrifuged (538 g, 10 min, 4 °C), and the pellet was resuspended with complete RPMI medium. The cell counts and viabilities were determined using an automated cell counter, after staining the cells with trypan blue (Countess^TM^, Invitrogen, Madrid, Spain), following usual laboratory procedures, as described in Reference [22]. The lymphocytes were immediately used to characterize their phenotype, and to determine their ability to proliferate and secrete cytokines.

### 2.7. Mesenteric Lymph Node Cells Stimulation and Proliferation Assay

MLNs cell activation was performed in sterile conditions, using 96-well tissue culture plates (TPP^®^, Trasadingen, Switzerland), which were pre-incubated with 200 μL/well of monoclonal antibodies (mAbs) anti-CD3 (10 µg/mL, BD Biosciences, San Diego, CA, USA) and anti-CD28 (2 µg/mL, BD Biosciences, San Diego, CA, USA) (2 h, 37 °C, 5% CO_2_). Then, MLNs lymphocyte suspensions (5 × 10^4^ cells/mL) were added to each well. A total of eight wells were used for each sample: four pre-incubated with the mitogenic mAbs (stimulated cells, SC) and four without pre-incubation (non-stimulated cells, NSC). The plates were incubated for 48 h, at 37 °C and 5% CO_2_. Four hours before the end time of the incubation, 5-bromo-2’-deoxyuridine (BrdU, 20 μL/well, Merck, Darmstadt, Germany) was added to measure its incorporation as an indicator of DNA synthesis in a colorimetric immunoassay.

The plates were centrifuged (210 g, 5 min) and supernatants were collected and stored at −80 °C, until cytokine analysis. After fixation, the anti-BrdU mAbs and peroxidase conjugated anti-BrdU mAbs and 3,3’,5,5’-tetramethylbenzidine (TMB) were added, following the manufacturer’s recommendations of the BrdU Cell Proliferation Assay Kit (Merck, Darmstadt, Germany). The absorbance (Abs) was measured at 450 nm (Multiskan MS, Labsystems, Helsinki, Finland). Data were expressed as a proliferation rate as follows: Proliferation rate = (A/B), where, A = ((Abs-SC − Abs-NSC)/Abs-NSC) _supplemented group_, and B = ((Abs-SC − Abs-NSC)/Abs-NSC) _reference group_.

### 2.8. Quantification of Cytokine Secretion by Mesenteric Lymph Node Lymphocytes

Supernatants collected after the cell stimulation process described above, were used for the quantification of IL-2, IL-4, IL-10, IL-13, IL-17A, interferon (IFN)-γ, and tumor necrosis factor (TNF)-α concentration using a ProcartaPlex^®^ Multiplex Immunoassay, according to the manufacturer’s instructions (eBioscience, San Diego, CA, USA) and as previously described in Reference [19]. Each analyte’s concentration was detected using the MAGPIX instrument (Luminex Corp., Austin, TX, USA), in the facilities of the Scientific and Technological Centers of the University of Barcelona (CCiT-UB), and the results were analyzed using xPONENT^®^ 4.2 software (Luminex Corp., Austin, TX, USA). The limits of detection were as follows: 2.10–8600 pg/mL for IL-2; 0.85–3500 pg/mL for IL-4; 14–55,700 pg/mL for IL-10; 3.17–13,000 pg/mL for IL-13; 2.61–2675 pg/mL for IL-17A; 4.35−17,800 pg/mL for IFN-γ; and 3.08−12,600 pg/mL for TNF-α.

### 2.9. Immunofluorescence Staining and Flow Cytometry Analysis

Lymphocytes from MLNs (5 × 10^4^ cells) were immunophenotyped by multiple immunofluorescence staining technique. Mouse anti-rat mAbs conjugated to fluorescein isothiocyanate (FITC), phycoerythrin (PE), peridinin-chlorophyll-a protein (PerCP), allophycocyanin (APC) or APC-cyanine(Cy)7 were used, as in previous studies [19]. For cell subset differentiation, five different mAbs panels were used; Panel 1: TCRαβ/NKR-P1A/CD8α; Panel 2: CD8α/CD8β/TCRγδ; Panel 3: αE integrin/CD62L/CD8α/CD4; Panel 4: CD45RA/TLR4/CD8α/CD4/CD25; and Panel 5: CD25/CD4/Foxp3. With the first panel, NK cells (NKR-P1A^+^ TCRαβ^−^), natural killer T (NKT) cells (NKR-P1A^+^ TCRαβ^+^), and TCRαβ^+^ cells (TCRαβ^+^ NKR-P1A^−^) could be differentiated, which in combination with the TCRγδ^+^ cells (obtained from Panel 2) constituted the total of T cells. B cells (CD45RA^+^) were identified with Panel 4. The mAbs used in this study are detailed in the Supplementary Materials (Table S1).

Briefly, cells were incubated with a mixture of 10 μL of saturating concentrations of each mouse anti-rat mAbs in PBS pH 7.2, containing 2% FBS and 0.1% sodium azide (Merck, Darmstadt, Germany), at 4 °C in darkness for 20 min. For T reg evaluation, an intracellular staining was performed. For that, cells previously labeled with anti-CD4-PE and anti-CD25-FITC mAbs, were fixed/permeabilized using a specific buffer kit (eBioscience, San Diego, CA, USA). Then, intracellular staining with anti-Foxp3-APC mAb was carried out, under the same conditions as extracellular staining. After washing, all stained cells were fixed with 0.5% paraformaldehyde (Panreac, Barcelona, Spain) and stored at 4 °C in darkness, until analysis by flow cytometry. For each sample, a positive control staining using each isotype matched mAbs and a negative control without staining were included. Analyses were performed in a Gallios™ Cytometer (Beckman Coulter, Miami, FL, USA) in the CCiT-UB. Data were assessed by FlowJo^®^ version 10 software (Tree Star Inc., Ashland, Covington, KY, USA). Results were expressed as percentages of positive cells in the lymphocyte population, selected according to their forward- and side-scatter characteristics (FSC/SSC) using previous studies protocols [19], or in a selected population. The gating strategy was specific for each panel used, but overall, markers (e.g., CD8α in Panel 1, αE integrin, and CD62L in Panel 3) were evaluated in specific gate subsets (e.g., NK, NKT, or T cells in Panel 1, and CD8 or CD4 cells in Panel 3).

### 2.10. Statistical Analysis

Statistical analyses were performed using the IBM Social Sciences Software Program (SPSS, version 22.0, Chicago, IL, USA). Levene’s and Shapiro−Wilk tests were applied to assess variance equality and normal distribution, respectively. When the results demonstrated equality of variance and normal distribution, a one-way analysis of variance (ANOVA) test was performed. Nonparametric tests were carried out when normal distribution and equality of variance did not exist. Specifically, Kruskal–Wallis and Mann–Whitney U tests were used to assess significance for independent samples. Significant differences were established at *p* < 0.05. Data in the text, tables, and figures are expressed as the mean ± standard error of the mean (S.E.M).

## 3. Results

### 3.1. Animal Growth

Body growth was assessed in the three groups receiving supplementation (TGF-β2, EGF, and FGF21), and compared with those receiving the vehicle (REF). Body weight increased during suckling period in the REF group (*p* < 0.05) and the supplementations did not affect this growth pattern (Supplementary Materials, Figure S1). With respect to morphometric variables and SI growth, there were no differences among the studied groups at any age considered (Table 1).

All groups increased BMI, SI relative weight, and SI relative length with age (*p* < 0.05, day 21 vs. day 14). Regarding the Lee index, only the animals from the REF group and those supplemented with TGF-β2, showed significant differences associated with age (*p* < 0.05), but the supplementation with EGF and FGF21 did not display such a significant age difference.

### 3.2. IgA and IgM Concentration in Gut Wash

Intestinal secretory IgA and IgM were determined at day 21 of life. At that age, the pups’ intestinal levels were not entirely influenced by maternal breast milk, as happens earlier in life when breast milk (very rich in IgA) is the only source of food. Nevertheless, none of the interventions (TGF-β2, EGF, and FGF21 supplemented groups) showed significant differences regarding IgA or IgM, compared to the REF group (Supplementary Materials, Figure S2).

### 3.3. Mesenteric Lymph Node Lymphocytes Composition

The proportion of the main lymphocyte subsets in MLN was established during (day 14) and at the end of (day 21) suckling period. On both days, most of the MLN cells (~75%) were T cells, the majority of them being TCRαβ^+^ cells and only ~3% TCRγδ^+^ cells; B cells counted for about 15–18%, NK and NKT cells represented subpopulations with less than 3% of total lymphocytes, and Treg cells were ~4% (Table 2).

Although at 14 days there were no differences among the supplemented groups, some differences could be seen at 21 days (Table 2). At the end of the suckling period, only the EGF supplementation showed a significant decrease in the percentage of TCRγδ^+^ cells, compared to the REF group (*p* < 0.05). Moreover, the REF and groups supplemented with TGF-β2 and EGF decreased the NK cell proportion, in comparison to the same group at 14 days (*p* < 0.05). The animals from the REF and FGF21 groups increased the percentage of NKT cells with age, and only those from the EGF group were able to decrease the proportion of TCRγδ^+^ and B cell percentages at day 21 (*p* < 0.05 vs. day 14).

**Figure 1:**
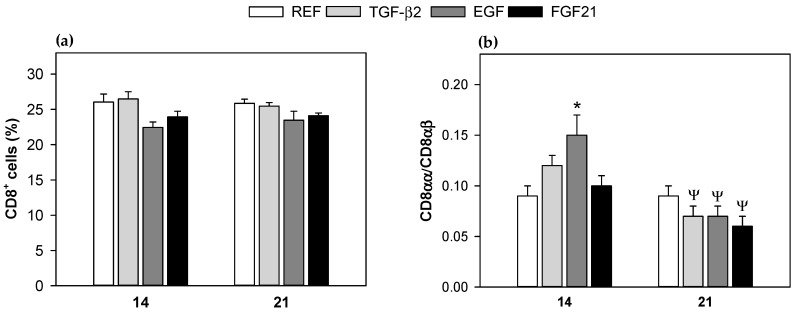
Proportion of CD8^+^ cells and ratio CD8αα/CD8αβ in mesenteric lymph nodes at 14 and 21 days of life. (**a**) % CD8^+^ cells; (**b**) The ratio CD8αα/CD8αβ was calculated as the quotient of the percentages of CD8αα cells and CD8αβ cells in CD8^+^ cells (%). Results are expressed as mean ± standard error of the mean (S.E.M) (*n* = 9). Statistical differences: * *p* < 0.05 vs. reference group (REF group) at same age; ^Ψ^ *p* < 0.05 vs. same group at day 14. TGF-β2: transforming growth factor-β2. EGF: epidermal growth factor. FGF21: fibroblast growth factor 21.

The percentage of CD8^+^ cells and the CD8αα/CD8αβ ratio from MLNs lymphocytes, were determined as indicators of immune maturation (Figure 1). The proportion of CD8^+^ cells did not show significant differences, either associated with age (day 14 vs. day 21) or to any of the supplementations, compared to the REF group (Figure 1a). However, regarding the results from CD8αα/CD8αβ ratio, the EGF supplementation induced a significant increase at 14 days (*p* < 0.05 vs. REF, Figure 1b). At day 21, in all supplemented groups, the CD8αα/CD8αβ ratio had decreased when they were compared to their respective group at day 14, but no differences among the interventions and the REF groups were found (Figure 1b).

**Figure 2:**
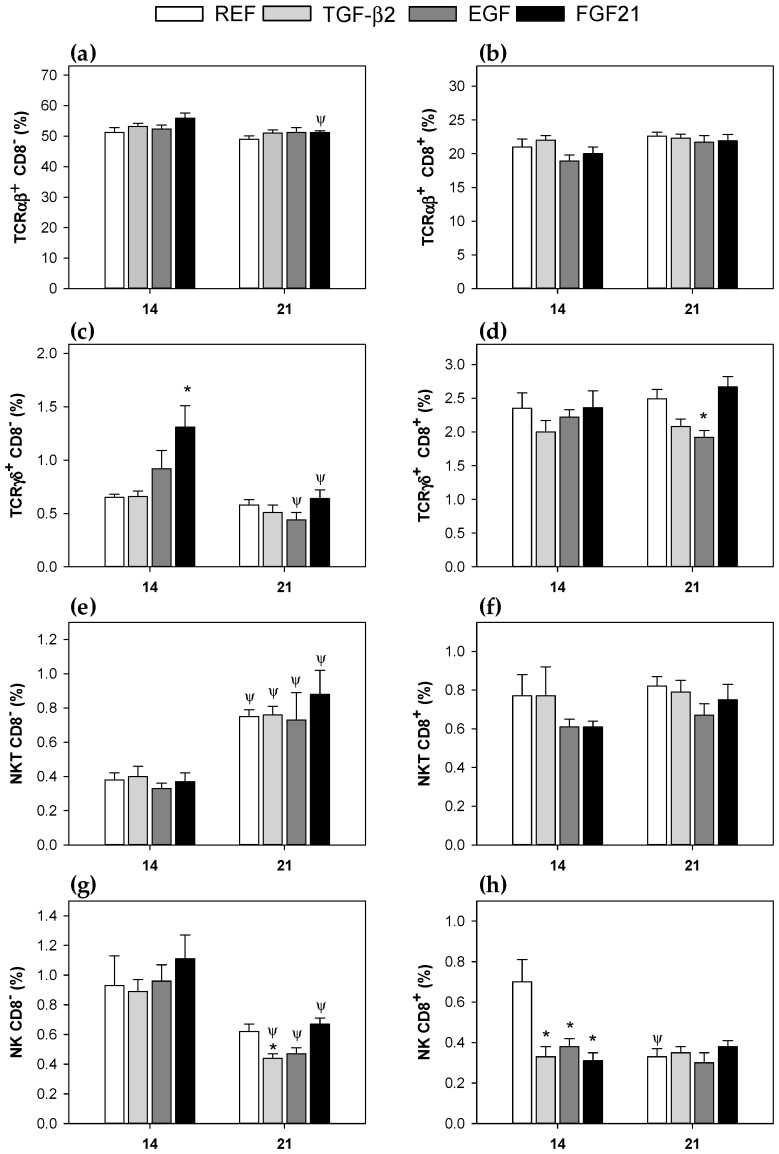
Percentages of CD8^+^ and CD8^−^ lymphocyte subsets in mesenteric lymph nodes at 14 and 21 days of life. (**a**) TCRαβ^+^ CD8^−^; (**b**) TCRαβ^+^ CD8^+^; (**c**) TCRγδ^+^ CD8^−^; (**d**) TCRγδ^+^ CD8^+^; (**e**) NKT CD8^−^; (**f**) NKT CD8^+^; (**g**) NK CD8^-^; and (**h**) NK CD8^+^. Results are expressed as mean ± S.E.M (*n* = 9). Statistical difference: * *p* < 0.05 vs. REF group at same age; ^Ψ^ *p* < 0.05 vs. same group at day 14.

Further analysis of CD8^+^ and CD8^−^ subsets revealed some changes associated with the supplementations (Figure 2).

Regarding the TCRαβ^+^ subsets (CD8^+^ and CD8^−^, respectively, Figure 2a,b), no changes due to supplementation were found either at 14 or at 21 days, with respect to the REF group. Only, the supplementation with FGF21 decreased the proportion of TCRαβ^+^ CD8^−^ (~8%), when values from day 21 were compared to day 14 (Figure 2a, *p* < 0.05). The TCRγδ^+^ CD8^−^ cell percentage (Figure 2c), increased due to supplementation with EGF and FGF21 at 14 days, with only the latter achieving statistical significance, but these changes were not observed at the end of the suckling period (day 21). However, both interventions showed a 50% decrease in the TCRγδ^+^ CD8^−^ cell proportion associated with age (*p* < 0.05, day 21 vs. day 14). Moreover, the TCRγδ^+^ CD8^+^ cell proportion in the EGF group (Figure 2d), was lower than the REF group at day 21 (*p* < 0.05). In relation to the NKT population (CD8^-^ and CD8^+^ subsets), only the NKT CD8^−^ subset showed changes associated with age for all groups (Figure 2e), but not due to the supplementations.

However, NKT CD8^+^ cell proportions were not affected (Figure 2f). Results from the NK cell population showed that in supplemented groups, there was a decrease in NK CD8^-^ cell proportions related to age (Figure 2g, *p* < 0.05, day 21 vs. day 14), and that only supplementation with TGF-β2 induced a significant decrease compared with the REF group at 21 days (Figure 2g, *p* < 0.05 vs. REF). The phenotype of the intestinal NK cells based on CD8 expression, could be considered as immature (NK CD8^+^) or more mature (NK CD8^−^). The three supplementations were able to decrease the proportion of NK cells expressing CD8 at 14 days (Figure 2h), which reached similar values to those from reference 21-day-old rats. Thus, only the REF group decreased its percentage of NK CD8^+^ cells at 21 days, with respect to day 14 (Figure 2h).

**Figure 3:**
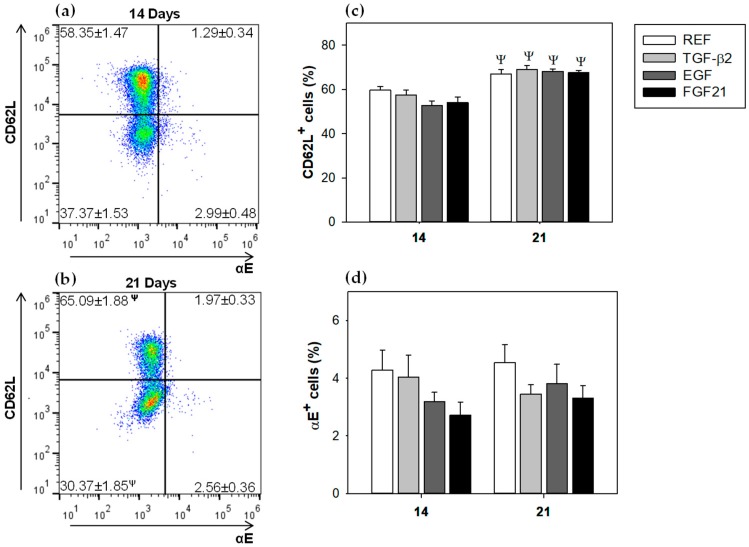
Surface expression of αE integrin and CD62L selectin, in mesenteric lymph nodes at 14 and 21 days of life. (**a**) CD62L/αE integrin surface expression at 14 days, (**b**) CD62L/αE integrin surface expression at 21 days, (**c**) CD62L^+^ cells (%) and (**d**) αE^+^ cells (%). Results are expressed as mean ± S.E.M (*n* = 9). Statistical difference: ^Ψ^ *p* > 0.05 vs. same group at day 14.

The lymphocytes’ commitment to the mucosal compartment were studied by means of the proportion of cells expressing two adhesion molecules of importance in the intestinal homing; thus, the total percentage of cells bearing the selectin CD62L and the αE integrin were determined (Figure 3).

Figure 3a,b show the molecular pattern of αE/CD62L on day 14 and 21. The CD62L molecule was expressed on both days in high proportion of cells (~60%). Although the percentage of CD62L^+^ cells increased significantly with age in all studied groups (Figure 3a–c, *p* < 0.05), none of the supplemented groups induced significant differences, when they were compared to the REF group at the same age. The αE^+^ cells were present in a proportion lower than 5% at both studied times (Figure 3b–d) and were not modified by any GFs administration (Figure 3d).

The percentages of integrin αE^+^ cells and selectin CD62L^+^ cells in CD8^+^, CD4^+^, and B cells were further studied (Table 3). None of the supplemented groups showed significant differences, compared to the REF group at the same age. All studied groups increased CD62L^+^ CD8^+^ and CD62L^+^ CD4^+^ percentages with age (*p* < 0.05, day 21 vs. day 14). The REF group increased (~15%) αE^+^ B cells subset proportion, with respect to the same group at 14 days, whereas all supplemented groups decreased (~20%) this subset with age (*p* < 0.05). In addition, the supplementation with EGF and FGF21, decreased the percentage of αE expression in CD4^+^ cells with age. Although there was a tendency to increase with age, the proportion of CD62L^+^ in B cells in all groups, it was only significant in the TGF-β2 group (*p* < 0.05).

At the end of the suckling period, activated CD4 cells (Foxp3^−^ CD25^+^ CD4^+^ cells) and Treg cells (Foxp3^+^ CD25^+^ CD4^+^ cells) were also studied, and no changes were found due to diets (day 21), with all groups together having a mean percentage of 1.22 ± 0.12 and 3.81 ± 0.11, respectively.

### 3.4. Proliferation and Cytokine Production by Mesenteric Lymph Nodes Cells

To determine the functional capacity of MLN lymphocytes, we studied their proliferative response and their ability to secrete cytokines on days 14 and 21. With regard to the proliferation of MLNs cells on days 14 and 21, GFs supplementations were not able to significantly modify such lymphocyte function induced by anti-CD3 and anti-CD28 mAbs (Supplementary Materials, Figure S3). However, cytokine production was influenced by GFs supplementation (Table 4).

The pattern of cytokines released from MLNs cells at day 14, showed a high secretion of IFN-γ, followed by IL-10. In the conditions tested, the secretion of IL-2, IL-4, IL-13, IL-17A, and TNF-α was lower than 90 pg/mL. IL-10/TNF-α (anti‑inflammatory/pro-inflammatory cytokines) and IFN-γ/IL-4 (Th1/Th2) ratios were also calculated. No changes in the cytokine pattern at day 14 were detected by GFs supplementation. However, at day 21, some changes appeared due to supplementations and due to age. IL-2 production was 5–10 times higher due to age in the REF, TGF-β2, and EGF groups (*p* < 0.05 day 21 vs. day 14). IL-4 levels were ~50% lower in the EGF group at day 21, than those at day 14 (*p* < 0.05). The groups that received EGF and FGF21 for 21 days, decreased the IL-10 content ~1.5 times with respect to their values at day 14 (*p* < 0.05). IL-13 secretion was lower at day 21 with respect to day 14, in the EGF and FGF21 groups (*p* < 0.05), and the three GFs groups showed lower values than those present in the REF group (*p* < 0.05). The lower levels of IFN-γ production at day 21 compared to day 14 were only significant in the REF group, which accounted for a 50% reduction (*p* < 0.05). Moreover, TNF-α release decreased (~30%) with age in the REF, TGF-β2, and FGF21 groups, whereas supplementation with EGF increased TNF-α production (*p* < 0.05 vs. REF group).

The IL‑10/TNF-α ratio decreased according to age in the EGF and FGF21 groups, and in the EGF group, values were significantly lower than the REF group (*p* < 0.05).

## 4. Discussion

At birth, the immune system is immature, as evidenced by a poor antibody production and low proliferative response of immune cells. In addition, there are mucosal immune impairments, such as low intestinal IgA content, reduced number of B lymphocytes in the intestinal mucosa, and few intestinal T cells [23]. Immune development is driven, among other factors, by components of breast milk. It is known that growth factors, such as TGF-β2 and EGF, regulate the immune response in early life and confer protective effects against gut mucosal inflammation by enhancing oral tolerance [1,2,7]. FGF21, which is also present in maternal milk, is a less-studied component and could be involved in such effects as well [16]. The current study aimed to evaluate whether supplementation with TGF-β2, EGF, or FGF21 had a role in immune maturation in early life. A rat pup model was used to evaluate the effect of a daily supplementation with TGF-β2, EGF, or FGF21 during suckling on the GALT, particularly in the MLNs, which is an inductor site of intestinal immune response. We assessed the immune maturation by functions, such as lymphoproliferation, cytokine production ability, and establishing lymphocyte MLNs composition.

Regarding growth, the body weight of pups was not modified due to supplementation with any of the GFs studied. These results were in line with other investigations, in which rats receiving either TGF-β2 [10] or EGF [24,25] during the first two weeks of life did not change their body weight. Likewise, although a study showed that the body weight of FGF21-knockout mice compared to wild-type 3-month-old mice was not different [26], its physiological role as a weight regulating factor was discussed [18]. Overall, it seems that the tested GFs, under the conditions we used, did not have a key role in the growth of neonates.

Intestinal length and weight are useful tools for evaluating the primary impact of a nutrient on the maturation of the rat small intestine [27]. The GFs supplementation in the current study did not affect these variables. However, it has been reported that suckling rats receiving intraperitoneal administration of EGF (100 μg/kg) for only two days, increased stomach and intestinal weights [24]; and that rat pups receiving oral administration of EGF through formula at concentrations exceeding the reported concentrations of EGF in rodent milk, enhanced intestinal growth (weight and length) [15]. Thus, in agreement with our results, only high doses of these compounds seem to be able to modify intestinal growth.

Although no impact on the body and intestinal growth due to any of these GFs was observed, some effects of TGF-β2, EGF, or FGF21 on immune variables have been shown to be specific; and therefore, the influence of each GF tested on MLN lymphocyte maturation will be discussed separately.

Transforming growth factor-β (TGF-β) has a wide range of biological activities and among them, it has an important role in cell proliferation and differentiation. TGF-β acts as a cytokine having predominantly suppressive effects on the growth of T and B lymphocytes. In this study, the supplementation of suckling rats with TGF-β2 did not have a significant influence on unspecific MLNs proliferative cell response. This result contrasted with a report showing that suckling rats receiving a whey-enriched TGF-β formula, down-regulated the MLNs lymphocyte proliferative response to specific antigen after being sensitized [28]. On the other hand, we observed that MLNs cells after TGF-β2 supplementation did not modify the changes in IL-2 and TNF-α secretion associated with age, but attenuated IL-13 production at day 21 with respect to reference animals. The attenuation on IL-13 production, a Th2 cytokine linked to allergic processes [29,30,31], agrees with results showing that Brown Norway rat pups receiving a formula with TGF-β2 between 4 and 18 days of life shifted the immune response from a Th2 type towards a Th1 profile [13]. Likewise, it is known that low levels of IL-13 in colostrum and mature milk are associated with less eczema in early life [32]. Thus, our results suggested that early TGF-β2 intake could play an important role in the prevention of Th2 mediated alterations, such as allergy.

In the current study, the TGF-β2 supplementation was already able to modulate MLN lymphocyte composition in suckling rats. It decreases the proportion of the immature phenotype of the intestinal NK cells (NK CD8^+^) on day 14 at levels observed later (day 21); which indicated a positive action on the intestinal immune maturation. This developmental pattern, decreasing CD8 expression in the NK cell surface with age, has previously described in rat neonatal intraepithelial lymphocytes (IEL) and MLN cells [19,27]. In line with this, we found that, although no changes in total CD8^+^ cells were observed due to TGF-β2 supplementation, a lower CD8αα/CD8αβ ratio at day 21, compared to day 14 appeared. This ratio has been described to be reduced according to age in healthy MLNs cells from suckling rats [19], thus our results may suggested a promotion of the intestinal immune system maturation. This contrasts with results in BALB/c mice pups, showing higher CD8^+^ T cell proportions when their lactating mothers were treated with mAbs, against TGF-β twice a week from delivery until weaning [33]. On the other hand, we did not find changes in the MLNs Treg (CD4^+^ CD25^+^ Foxp3^+^) cell proportion. However, some authors have suggested that oral tolerance induced by breastfeeding could depend on TGF-β signaling from breast milk, which would be able to up-regulate Foxp3^+^ cells [33].

Regarding the intestinal humoral immune response, it is known that IgA is poorly produced by the neonate mucosal immune system [1,28], and that TGF-β1 and TGF-β2 from mammalian milk are able to stimulate the synthesis of mucosal IgA [28,33]. Thus, TGF-β acting in synergy with IL-10, can promote IgA production and oral tolerance induction [28]. However, our results did not show significant changes in the intestinal IgA and IgM content of the suckling rats. This lack of effect could be due to the fact that breast milk contains IgA and IgM, which are transferred to the pups, and then these milk antibodies in the intestine would mask the pups’ own levels. For this reason, intestinal IgA and IgM assessment cannot be a good marker of nutritional supplementation at this level.

Epidermal growth factor (EGF) is a peptide that modulates a variety of biological responses, such as cell proliferation and differentiation [14]. It is known that it has a clear effect on epithelium, where it accelerates maturation and stimulates cell proliferation [34,35,36]. However, in the current study, we have not detected any effect of the EGF supplementation on the proliferative response of MLNs cells in the suckling pups. There is evidence that EGF prevents and reduces the incidence and severity of NEC by modulating important transcription factors for cytokine regulation [37], and it is known that the balance of pro-inflammatory and anti-inflammatory cytokines may play a key role in the development of NEC [17]. This effect may be attributable to a down-regulation of pro-inflammatory IL-18 and to an increase of anti-inflammatory IL-10 at the site of injury [17,37]. Here we found that, 21-day EGF supplementation increased TNF-α and decreased IL-13 production by MLNs cells. The changes in these two cytokines, contained in maternal milk [32,38], could be associated with positive effects. Indeed, decreased IL-13 can be useful in the prevention of allergy, as stated for TGF-β2 [29,30,31]. Moreover, EGF supplementation kept the levels of TNF-α on day 21 at the same level as that on day 14, which could play in favor of its own effects because it is known that EGF receptor (EGF-R) is up-regulated by TNF-α [39]. Regarding MLNs cell composition, EGF supplementation shares some maturate effects with TGF-β2, such as the induction of lower levels of NK CD8^+^ cells and CD8αα/CD8αβ ratio.

Finally, focusing on fibroblast growth factor 21 (FGF21), recent studies showed that its function is not limited to the regulation of metabolism, but that it is also involved in the protection of multiple physiological processes, such as oxidation, inflammation, atherosclerosis, and aging processes [40]. A recent study demonstrated that, in the spleen of mice with collagen-induced arthritis (CIA), FGF21 reduces the expression of inflammatory cytokines, such as IL-17, TNF-α, IL-1β, IL-6, IL-8, and MMP3, whereas IL-10 levels were increased, compared to PBS treated CIA mice [41]. In line with this, we have not found any significant difference with respect to non-supplemented animals on MLNs inflammatory cytokines, but the FGF21 supplementation decreased IL-13 at 21 days, as we also found with the other GFs, suggesting, therefore, its role in preventing allergic events.

It is known that FGF21 is present in breast milk and does not contribute to systemic levels in mouse neonates, but appears to act locally on the mouse neonatal intestine [16]; and it is unknown whether this factor plays an important role in the maturation of the immune system. We found that suckling rats receiving FGF21 promoted maturation of the early life intestinal immune system by accelerating the decrease in the proportion of NK CD8^+^ cells and the decrease in the CD8αα/CD8αβ cell ratio in MLNs, as well as TGF-β2 and EGF. This is the first time that the immunomodulatory effect of FGF21 has been demonstrated in early life.

## 5. Conclusions

In conclusion, our study demonstrated that supplementation with TGF-β2, EGF, or FGF21 during the suckling period had an immunoregulatory effect. Although some specific effects appeared, the three growth factors were able to modulate similar aspects of MLN cells, such as promoting lymphocyte maturation, as observed by increasing NK cells with a more mature phenotype (CD8^−^) and reducing IL-13 production, which could be useful in avoiding allergic processes. Further studies should be carried out to establish the effect of the supplementation of TGF-β2, EGF, or FGF21 on the response of suckling pups in other parts of the intestinal immune system, such as in the intestinal epithelium or even at the systemic level.

## Figures and Tables

**Table 1:** Morphometric variables and small intestinal growth.

		BMI	Lee Index	SI Relative Weight	SI Relative Length
		(g/cm^2^)	((∛g/cm) × 1000)	(%)	(%)
Day 14	REF	0.365 ± 0.009	332.9 ± 3.473	3.51 ± 0.05	118.29 ± 5.49
	TGF-β2	0.373 ± 0.010	336.9 ± 3.844	3.59 ± 0.14	114.34 ± 4.28
	EGF	0.340 ± 0.007	327.0 ± 2.845	3.48 ± 0.06	122.64 ± 2.91
	FGF21	0.371 ± 0.009	337.4 ± 4.044	3.18 ± 0.06	105.59 ± 5.31
Day 21	REF	0.404 ± 0.010 ^Ψ^	319.5 ± 2.551 ^Ψ^	4.15 ± 0.24 ^Ψ^	89.57 ± 3.12 ^Ψ^
	TGF-β2	0.404 ± 0.009 ^Ψ^	320.8 ± 3.225 ^Ψ^	4.25 ± 0.09 ^Ψ^	85.65 ± 2.20 ^Ψ^
	EGF	0.404 ± 0.007 ^Ψ^	322.9 ± 2.574	4.38 ± 0.07 ^Ψ^	88.34 ± 2.20 ^Ψ^
	FGF21	0.420 ± 0.011 ^Ψ^	329.0 ± 4.053	4.78 ± 0.17 ^Ψ^	89.97 ± 4.01 ^Ψ^

BMI: body mass index. SI: small intestine. TGF-β2: transforming growth factor-β2. EGF: epidermal growth factor. FGF21: fibroblast growth factor 21. Results are expressed as mean ± standard error of the mean (S.E.M) (*n* = 9). ^Ψ^ *p* < 0.05 vs. day 14 at same group.

**Table 2:** Main lymphocyte subsets in mesenteric lymph nodes.

		Reference	TGF-β2	EGF	FGF21
Day 14	T cells (%)	75.90 ± 1.80	77.90 ± 1.39	74.36 ± 1.88	79.60 ± 1.50
	T TCRαβ^+^ (%)	72.90 ± 1.98	75.24 ± 1.29	71.22 ± 1.94	75.90 ± 1.20
	T TCRγδ^+^ (%)	3.00 ± 0.20	2.66 ± 0.20	3.14 ± 0.23	3.70 ± 0.40
	NK (%)	1.63 ± 0.30	1.22 ± 0.08	1.35 ± 0.12	1.42 ± 0.17
	NKT (%)	1.15 ± 0.14	1.18 ± 0.20	0.94 ± 0.05	0.98 ± 0.06
	B cells (%)	17.65 ± 1.65	16.19 ± 1.40	19.62 ± 1.23	15.86 ± 1.07
Day 21	T cells (%)	74.60 ± 1.36	75.86 ± 1.10	75.29 ± 1.20	76.41 ± 0.69
	T TCRαβ^+^ (%)	71.57 ± 1.44	73.28 ± 1.14	72.93 ± 1.26	73.10 ± 0.77
	T TCRγδ^+^ (%)	3.07 ± 0.15	2.58 ± 0.15	2.36 ± 0.13 *^,Ψ^	3.31 ± 0.13
	NK (%)	0.95 ± 0.07 ^Ψ^	0.79 ± 0.06 ^Ψ^	0.77 ± 0.08 ^Ψ^	1.05 ± 0.07
	NKT (%)	1.57 ± 0.07 ^Ψ^	1.55 ± 0.11	1.39 ± 0.21	1.63 ± 0.18 ^Ψ^
	B cells (%)	14.67 ± 0.71	14.30 ± 0.64	12.95 ± 1.19 ^Ψ^	13.23 ± 0.62

Results are expressed as mean ± S.E.M (*n* = 9). * *p* < 0.05 vs. reference (REF) group at same age; ^Ψ^ *p* < 0.05 vs. day 14 at same group.

**Table 3:** Surface expression of the αE integrin and the CD62L selectin in CD4^+^, CD8^+^, and B cells in mesenteric lymph nodes.

		**14 Days**			
		**Reference**	**TGF-β2**	**EGF**	**FGF21**
%αE	CD8^+^	2.75 ± 0.72	2.92 ± 0.49	2.94 ± 0.52	2.88 ± 0.44
	CD4^+^	2.81 ± 0.39	3.07 ± 0.45	4.46 ± 0.34	3.80 ± 0.26
	B cells	15.98 ± 1.61	18.33 ± 1.39	18.92 ± 2.50	20.32 ± 3.12
%CD62L	CD8^+^	65.27 ± 1.56	62.38 ± 3.47	59.25 ± 2.52	59.21 ± 3.55
	CD4^+^	59.55 ± 1.76	55.41 ± 2.36	53.61 ± 1.37	53.65 ± 2.40
	B cells	48.43 ± 2.77	46.6 ± 2.05	38.77 ± 2.08	37.29 ± 4.78
		**21 Days**			
		**Reference**	**TGF-β2**	**EGF**	**FGF21**
%αE	CD8^+^	3.84 ± 0.48	2.98 ± 1.04	3.70 ± 0.48	3.97 ± 0.66
	CD4^+^	2.83 ± 0.75	2.61 ± 0.52	1.94 ± 0.18 ^Ψ^	2.52 ± 0.30 ^Ψ^
	B cells	18.43 ± 1.83 ^Ψ^	13.03 ± 1.55 ^Ψ^	14.95 ± 1.69 ^Ψ^	15.75 ± 1.16 ^Ψ^
%CD62L	CD8^+^	71.49 ± 1.38 ^Ψ^	72.10 ± 1.93 ^Ψ^	70.78 ± 2.19 ^Ψ^	72.88 ± 2.37 ^Ψ^
	CD4^+^	66.02 ± 1.74 ^Ψ^	68.24 ± 1.64 ^Ψ^	66.66 ± 0.42 ^Ψ^	66.80 ± 1.22 ^Ψ^
	B cells	56.79 ± 2.25	60.92 ± 2.17 ^Ψ^	60.82 ± 3.92	57.61 ± 2.37

Results are expressed as mean ± S.E.M (*n* = 9). ^Ψ^ *p* < 0.05 vs. day 14 at same group.

**Table 4:** Cytokine production by mesenteric lymph node cells after in vitro stimulation.

	**14 Days**			
**(pg/mL)**	**Reference**	**TGF-β2**	**EGF**	**FGF21**
IL-2	42.71 ± 5.96	39.24 ± 5.66	50.45 ± 6.66	56.72 ± 11.04
IL-4	89.82 ± 13.00	57.14 ± 7.81	82.65 ± 12.47	74.97 ± 12.18
IL-10	879.82 ± 239.40	688.01 ± 145.14	1450.87 ± 296.29	1326.99 ± 217.06
IL-13	25.38 ± 5.64	17.53 ± 3.03	31.91 ± 4.28	26.18 ± 4.03
IL-17A	71.98 ± 26.92	70.22 ± 20.16	60.12 ± 13.61	50.82 ± 9.53
IFN-γ	10323.26 ± 2391.46	8302.75 ± 1964.09	9329.17 ± 1662.87	7882.51 ± 2810.34
TNF-α	8.76 ± 0.98	9.48 ± 0.68	9.19 ± 1.16	7.40 ± 0.25
IL-10/TNF-α	115.00 ± 33.11	79.71 ± 21.80	170.87 ± 43.10	178.05 ± 29.25
IFN-γ/IL-4	110.35 ± 20.65	195.32 ± 61.39	124.85 ± 37.55	103.80 ± 26.93
	**21 Days**			
**(pg/mL)**	**Reference**	**TGF-β2**	**EGF**	**FGF21**
IL-2	390.57 ± 141.49 ^Ψ^	383.80 ± 89.21 ^Ψ^	244.79 ± 80.37 ^Ψ^	270.16 ± 114.54
IL-4	70.52 ± 10.51	52.21 ± 7.69	48.52 ± 1.91 ^Ψ^	66.60 ± 8.80
IL-10	705.45 ± 63.95	514.70 ± 55.30	508.26 ± 61.43 ^Ψ^	542.36 ± 78.82 ^Ψ^
IL-13	19.51 ± 2.44	12.92 ± 0.74 *	9.91 ± 1.24 *^,Ψ^	9.83 ± 1.65 *^,Ψ^
IL-17A	59.97 ± 7.14	46.91 ± 4.03	52.30 ± 10.7022	41.24 ± 11.77
IFN-γ	4231.26 ± 365.41 ^Ψ^	4488.50 ± 416.14	6900.74 ± 1129.78	4595.11 ± 1058.87
TNF-α	6.63 ± 0.11 ^Ψ^	6.55 ± 0.20 ^Ψ^	10.02 ± 0.40 *	5.91 ± 0.38 ^Ψ^
IL-10/TNF-α	106.47 ± 9.73	80.13 ± 9.55	50.48 ± 5.72 *^,Ψ^	95.64 ± 17.03 ^Ψ^
IFN-γ/IL-4	64.25 ± 9.61	111.99 ± 25.23	147.25 ± 27.9166	81.50 ± 25.37

Results are expressed as mean ± S.E.M (*n* = 9). * *p* < 0.05 vs. REF group at same age; ^Ψ^ *p* < 0.05 vs. day 14 at same group.

## Supplementary Materials

The following are available online at http://www.mdpi.com/2072-6643/10/9/1171/s1, Figure S1: Growth curve of all studied groups during the suckling period (from 1 to 21 days of life), Figure S2: IgA and IgM concentration in gut wash from 21-day-old animals after the different nutritional interventions, Figure S3: Proliferation of mesenteric lymph nodes lymphocytes at 14 and 21 days of life, Table S1: List of monoclonal antibodies (mAbs) used for immunophenotyping the mesenteric lymph nodes (MLN) cells.
