# Supplementary material for: TGF-β2, EGF, and FGF21 Growth Factors Present in Breast Milk Promote Mesenteric Lymph Node Lymphocytes Maturation in Suckling Rats
Source: Nutrients. 2018 Aug 27;10(9):1171. doi: 10.3390/nu10091171 (PMC6163676; doi:10.3390/nu10091171)
Supplement: Supplementary file 1 [file nutrients-10-01171-s001.pdf]

Supplementary Figure S1.

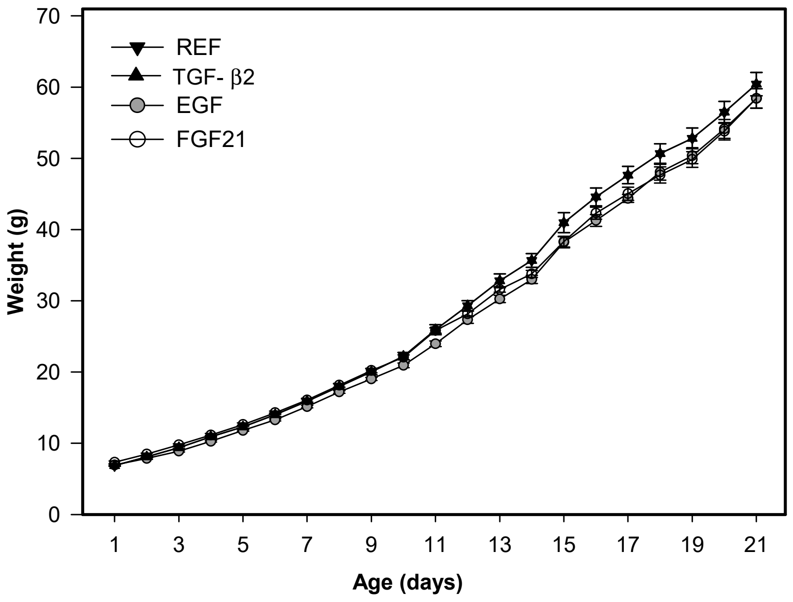

Growth curve of all studied groups during the suckling period (from 1 to 21 days of life). Results are expressed as mean ± S.E.M (n=9-27 pups/group).

Supplementary Figure S2.

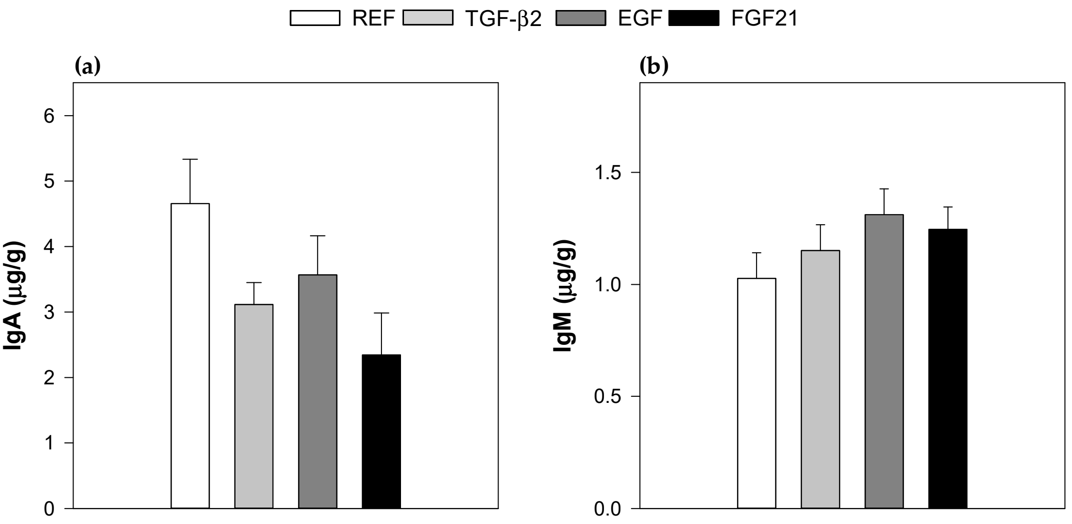

IgA and IgM concentration in gut wash from 21-day-old animals after the different nutritional interventions. **(a)** IgA; **(b)** IgM. Results are expressed as concentration of Igs normalized per gram of tissue used for the gut wash (µg/g) represented as mean ± S.E.M (n=9).

Supplementary Figure S3.

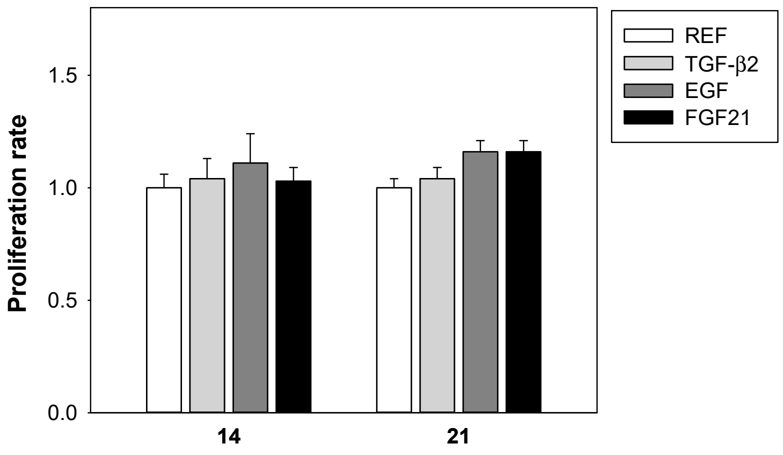

Proliferation of mesenteric lymph nodes lymphocytes at 14 and 21 days of life. Results are expressed as proliferation rate ± S.E.M (n=9).

Supplementary Table S1.

Table S1. List of mAbs used for immunophenotyping the MLN cells.

Anti-rat mAbs	Clone	Fluorochrome[1]	Concentration (μg/mL)	Supplier
Anti-CD4	OX-35	APC/PE	7/5	BD Biosciences
Anti-CD8α	OX-8	PerCP	10	BD Biosciences
Anti-CD8β	3.41	FITC	17	BD Biosciences
Anti-TCRαβ	R73	FITC	17	BD Biosciences
Anti-TCRγδ	V65	PE	10	BD Biosciences
Anti-NKR-P1A	10/78	PE	12	BD Biosciences
Anti-CD45RA	OX-33	APC-Cy7	20	BD Biosciences
Anti-CD25	OX-39	FITC	25	BD Biosciences
Anti-CD103 (αE)	OX-62	FITC	50	Biolegend
Anti- CD62L	OX-85	PE	13	Biolegend
Anti-TLR4	76B357.1	PE	25	Novus Biologicals
Anti-Foxp3	FJK-165	APC	5	eBioscience

[1]Allophycocyanin (APC), phycoerythrin (PE), peridinin-chlorophyll-a protein (PerCP), fluorescein isothiocyanate (FITC), APC-cyanine 7 (APC-Cy7)
